# Delayed cell death associated with mitotic catastrophe in γ-irradiated stem-like glioma cells

**DOI:** 10.1186/1748-717X-6-71

**Published:** 2011-06-10

**Authors:** Elke Firat, Simone Gaedicke, Chizuko Tsurumi, Norbert Esser, Astrid Weyerbrock, Gabriele Niedermann

**Affiliations:** 1 Department of Radiation Oncology, University Hospital Freiburg, Freiburg, Germany; 2 ProQinase GmbH, Freiburg, Germany; 3 Department of Neurosurgery, University Hospital Freiburg, Freiburg, Germany

## Abstract

**Background and Purpose:**

Stem-like tumor cells are regarded as highly resistant to ionizing radiation (IR). Previous studies have focused on apoptosis early after irradiation, and the apoptosis resistance observed has been attributed to reduced DNA damage or enhanced DNA repair compared to non-stem tumor cells. Here, early and late radioresponse of patient-derived stem-like glioma cells (SLGCs) and differentiated cells directly derived from them were examined for cell death mode and the influence of stem cell-specific growth factors.

**Materials and methods:**

Primary SLGCs were propagated in serum-free medium with the stem-cell mitogens epidermal growth factor (EGF) and fibroblast growth factor-2 (FGF-2). Differentiation was induced by serum-containing medium without EGF and FGF. Radiation sensitivity was evaluated by assessing proliferation, clonogenic survival, apoptosis, and mitotic catastrophe. DNA damage-associated γH2AX as well as p53 and p21 expression were determined by Western blots.

**Results:**

SLGCs failed to apoptose in the first 4 days after irradiation even at high single doses up to 10 Gy, but we observed substantial cell death later than 4 days postirradiation in 3 of 6 SLGC lines treated with 5 or 10 Gy. This delayed cell death was observed in 3 of the 4 SLGC lines with nonfunctional p53, was associated with mitotic catastrophe and occurred via apoptosis. The early apoptosis resistance of the SLGCs was associated with lower γH2AX compared to differentiated cells, but we found that the stem-cell culture cytokines EGF plus FGF-2 strongly reduce γH2AX levels. Nonetheless, in two p53-deficient SLGC lines examined γIR-induced apoptosis even correlated with EGF/FGF-induced proliferation and mitotic catastrophe. In a line containing CD133-positive and -negative stem-like cells, the CD133-positive cells proliferated faster and underwent more γIR-induced mitotic catastrophe.

**Conclusions:**

Our results suggest the importance of delayed apoptosis, associated mitotic catastrophe, and cellular proliferation for γIR-induced death of p53-deficient SLGCs. This may have therapeutic implications. We further show that the stem-cell culture cytokines EGF plus FGF-2 activate DNA repair and thus confound *in vitro *comparisons of DNA damage repair between stem-like and more differentiated tumor cells.

## Background

According to the tumor stem cell hypothesis, resistance to conventional therapies may reside in a subset of tumor cells with stem-like characteristics [1-3]. These cells are called cancer stem cells (CSCs) or cancer stem-like cells and are endowed with long-term self-renewal and a certain differentiation capacity. Several reports suggest that CSCs are indeed more resistant to standard chemo- and radiation therapy than non-CSCs [4-13]. However, most studies addressing cell death modalities have focused on apoptosis early after the genotoxic insult [6,9-12]. The importance of mitotic catastrophe as cause of cell death induced by genotoxic treatments has so far not been addressed in CSCs. Mitotic catastrophe is caused by altered mitoses and/or irreparable chromosome damage and is accompanied by micronucleation and multinucleation. Mitotic catastrophe causes a delayed mitosis-linked cell death and finally leads to apoptosis or necrosis [14-17].

Several explanations have been proposed for the higher gamma (γ)-ionizing radiation (IR) resistance of CSCs compared to non-CSCs: a stronger activation of DNA damage checkpoints associated with more proficient DNA damage repair [6], less initial DNA damage due to lower levels of γIR-induced oxidative radicals [7,13], as well as activation of stemness pathways [7,8]. However, compared to conventional glioblastoma cell lines, glioblastoma CSCs were either more radiosensitive and repaired γIR-induced DNA-double strand breaks (DSBs) less efficiently [18] or showed no difference in radio- and chemotherapy-induced DNA damage and repair [19,20]. Thus, the differences between CSCs and non-CSCs in γIR-induced DNA damage, damage repair and cell death are not fully clear.

We established cultures of immature stem-like cells from primary glioblastomas. Removal of the stem cell culture cytokines epidermal growth factor (EGF) and fibroblast growth factor-2 (FGF-2) and addition of fetal bovine serum (FBS) led in some but not all cases to differentiation of these stem-like cells. Using such directly related cultures, we examined the radioresponse of stem-like glioma cells (SLGCs) and of more differentiated glioma cells in terms of cell death mechanisms, focusing on both apoptosis and mitotic catastrophe. We also assessed whether the stem cell culture cytokines EGF and FGF-2 contribute to differences between stem-like and more differentiated tumor cells in terms of DNA damage levels and of apoptosis resistance upon γ-irradiation.

## Materials and methods

### Tumor samples and cell culture

Brain tumor samples were obtained following approval by the University of Freiburg ethical board (application number: 349/08) and informed written consent of patients. All patients were diagnosed as classical primary GBM. Tumors were dissociated into single cells with "Liberase Blendzymes" (Roche) for 45 min at 37°C. Cells were then allowed to form spheres in suspension culture in serum-free Neurobasal medium (Gibco) supplemented with EGF/FGF-2 (20 ng/ml each), B27, non-essential amino acids, penicillin/streptomycin, glutamax and heparin, on low attachment plates (Corning). For experiments, the cultures were expanded in plates coated with ECM proteins (mouse sarcoma-derived ECM, Sigma). The CSC-like properties were confirmed with serial neurosphere assays and serial xenotransplantation assays in BALB/c nude or non-obese diabetic/severe combined immunodeficient mice which were performed in accordance with protocols specifically approved by the animal care committee of the Regierungspräsidium Freiburg (registration number: G-10/64). Two SLGC cultures (G179 and G166) have previously been described by Pollard *et al*. [21] and were purchased from Biorep (Milan, Italy). For differentiation, the SLGCs were either transferred to DMEM supplemented with 10% FCS, penicillin and streptomycin, L-glutamine, non-essential amino acids and β-mercaptoethanol or to Neurobasal medium without EGF and FGF, supplemented with all-*trans*-retinoic acid (Sigma).

### γ**-irradiation**

Irradiations were performed using a Gammacell 40 ^137^Cs laboratory irradiator.

### Cell Growth and Viability Assay

An aliquot of cell suspension was mixed with Trypan blue solution (0.4% in PBS; Sigma), and the numbers of live and dead cells (viable cells excluded the dye and were unstained, nonviable cells were blue) were counted under a microscope.

### Apoptosis assays

Exponentially growing cells that had been seeded 24-60 h before were irradiated, and at the time points indicated stained with Annexin V and propidium iodide (PI) using an Annexin V-FITC Kit from Milteniy Biotec. Apoptosis was measured by flow cytometry on a Cytomics FC 500 instrument from Beckman Coulter.

### Assessment of mitotic catastrophe

24 to 48 h after seeding, cells were irradiated and, at the time points indicated, fixed and stained with 4'-6-diamidino-2-phenylindole (DAPI) for chromosome analysis under an Olympus BX41 fluorescence microscope equipped with a digital camera CC-12 soft imaging system (U-CMAD3, Olympus). For each assessment of the extent of mitotic catastrophe 200 nuclei were examined.

### Immunofluorescence staining

Cells grown on slides were fixed with Histofix for 15 min at room temperature. Thereafter, the cells were permeabilized with 0.2% Triton-X100. After blocking (with 2% bovine serum albumin and 5% goat serum in PBS for 1 h at room temperature), the cells were incubated with primary antibodies against one of the following proteins: Sox2 (Abcam), CD133 (Milteniy), GFAP (Dako), nestin, Tuj, or musashi (Chemicon) at 4°C for 1 h or overnight, followed by incubation with Alexa Fluor 488-labeled secondary antibodies (Invitrogen) for 20 min at room temperature. Nuclei were counterstained with DAPI, and cells analyzed using a BX41 fluorescence microscope (Olympus) equipped with the digital camera CC-12 soft imaging system U-CMAD3 at 100-fold magnification. CD133+ cells were isolated from CSC cultures with magnetic beads coated with CD133 antibody (Milteniy).

### Western blot analyses

Cell lysates were prepared in RIPA lysis buffer supplemented with protease inhibitor cocktail (Complete from Roche) and phosphatase inhibitors NaF and 7 Na_3_VO_4 _(Sigma). The blots were probed with the indicated antibodies and developed by enhanced chemiluminescence (Amersham Biosciences). The following antibodies were used: Sox2 (Abcam), musashi (Chemicon), nestin, γH2AX, p53, phospho-p53, Bcl-2, Bcl-xL, Mcl-1 and p21 from Cell Signaling, DNA-PK (BD Pharmingen), phospho-DNA-PK (Abcam), as well as actin and Bax (Santa Cruz). Quantification of signals was performed using Image Quant TL (Amersham Bioscience).

### Blocking the EGF and the FGF2 pathway

The binding of cytokines was blocked at the receptor level with monoclonal antibodies. The anti-EGFR antibody Cetuximab (Erbitux^®^; Merck KGaA, Darmstadt) was used at a concentration of 60 nM and the anti-FGFR1 monoclonal antibody (clone VBS1, Chemicon) at a concentration of 5 μg/ml. The antibodies were added 1 h prior to adding the cytokines.

### Cell surface marker determination by flow cytometry

Directly-PE-labeled antibodies against an extracellular glycosylation-dependent epitope (AC133) of CD133 (Milteniy) were used.

### Cell cycle analyses

Exponentially growing cells seeded 60 h before were irradiated, fixed at the indicated time points with 70% ethanol, and stored overnight at -20°C. Cells were then washed and incubated with PI (50 μg/mL) and RNase (100 μg/mL) for 2 h at 4°C. After washing, the cells were analyzed for DNA content by flow cytometry.

### Statistical analyses

All data are presented as mean ± SD and analyzed by Student's t test, two-tailed, with unequal variance. P < 0.05 was considered significant.

## Results

### Establishing cultures of stem-like and directly derived differentiated glioma cells

**Figure 1:**
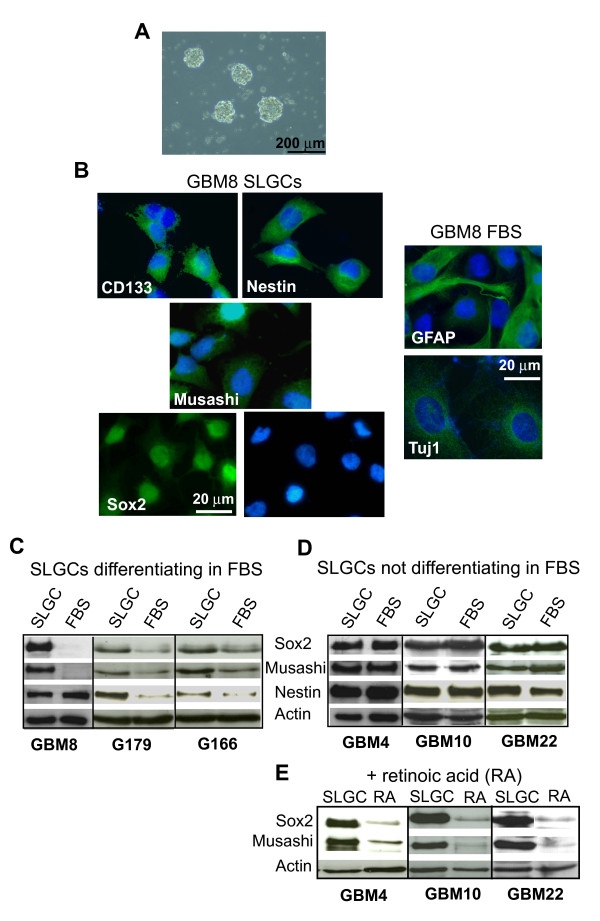
**Characterization of SLGCs and FBS cultures derived directly from the SLGCs**. **A**. Sphere formation 14 days after seeding 500 cells/well in 24 well plates. **B**. Immunofluorescence analysis of neural stem- and progenitor markers (Sox2, CD133, musashi, nestin) and differentiation markers (GFAP, Tuj) in SLGC and in differentiating FBS cultures. Nuclei were counterstained with DAPI. **C-E**. Western blot analysis of stem- and progenitor markers of SLGCs differentiating in FBS-containing medium (**C**), of SLGCs resistant to differention in FBS-containing medium (**D**) but differentiating after exposure to vitamin A (**E**). Blots shown are representative of at least three independent experiments. The analyses were performed after culturing for 4 weeks under differentiating conditions.

AC133/CD133 is an established CSC marker for glioblastoma [22]. However, the epitope is not detected in all glioblastomas; the AC133/CD133-negative population also contains CSCs, perhaps even the most primordial ones, and no surface markers are known for these types of cells [23-26], (Additional file 1). We therefore enriched immature glioma cells by culturing single cell suspensions of freshly resected glioblastomas in serum-free medium supplemented with EGF and FGF-2 to favor the growth of undifferentiated cells [27]. When cultured on low-attachment surfaces, these cells formed spheres (Figure 1A). The spheres were capable of generating new spheres under limiting passage conditions consistent with self-renewal (not shown). For large-scale propagation of undifferentiated cells we turned to monolayer culturing on extracellular matrix (ECM) proteins [21,28]. Alongside our own primary cultures (GBM8, GBM4, GBM10, and GBM22), we used the recently published primary SLGC lines G179 and G166, which also were raised by adherent culturing [21]. The cultures used were tumorigenic in immunocompromised mice ([21], and data not shown).

After a few passages, we maintained half the cells under stem cell conditions and exposed the other half to FBS without EGF and FGF, conditions widely used for *in vitro *differentiation of stem-like cells [6,11,18,21]. In some cases (GBM8, G179, G166), we observed differences in the morphology and changes in protein expression compatible with loss of stem cell phenotype and with differentiation (Figure 1B-D). Differentiating cells became larger and lost expression of stem and progenitor markers (Sox2, musashi, and nestin), instead expressing differentiation markers (e.g., GFAP, Tuj-1). Nestin expression, however, was not always eliminated, indicating abnormal differentiation.

Some SLGC lines (e.g., lines 4, 10, and 22) showed strong resistance against differentiation in FBS culture. However, all lines differentiated upon exposure to vitamin A (Figure 1E).

### Resistance to γIR-induced apoptosis in SLGC cultures early after irradiation

**Figure 2:**
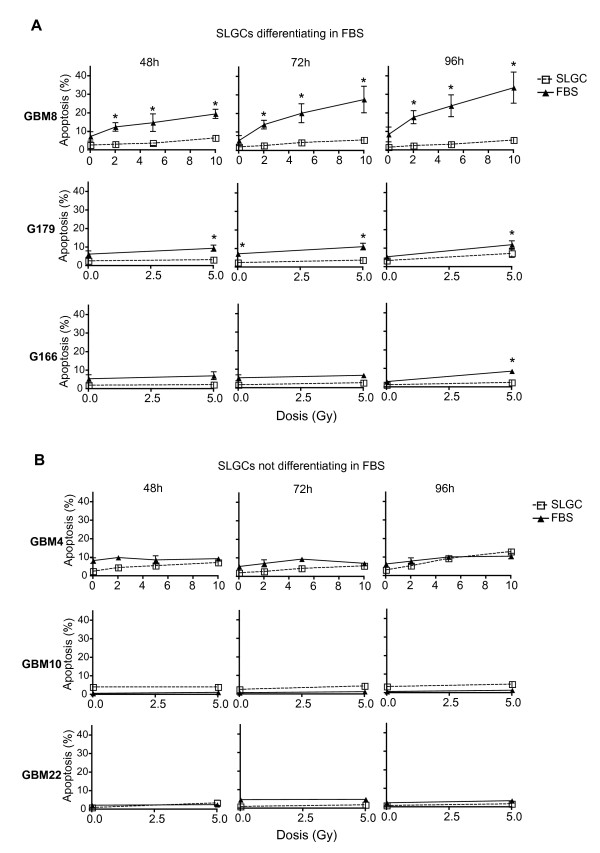
**Apoptosis resistance of SLGCs early after irradiation**. **A**. γIR-induced apoptosis of SLGCs and of derived cultures differentiating in FBS. **B**. γIR-induced apoptosis of SLGCs resistant to differentiation in FBS. Exponentially growing cultures were irradiated with the doses indicated and apoptosis was assessed flow cytometrically by measuring the binding of annexin-V and incorporation of PI 48, 72, and 96 h after irradiation. Mean ± S.D. of at least three experiments is shown; statistical significance (p < .05).

Apoptosis can occur immediately after irradiation as interphase death ("fast apoptosis"), after G2 arrest, or after one or several cell divisions ("late apoptosis") [29]. To determine susceptibility to γIR-induced apoptosis, both SLGC and FBS cultures were irradiated with 2, 5 or 10 Gy or sham-irradiated. 2 Gy is the daily dose in conventional fractionated radiotherapy; higher doses of 5 Gy and 10 Gy are used in hypofractionated treatments [30]. As in other studies assessing apoptosis in genotoxically treated CSCs [6,9,10,12], we first focused on apoptosis early after genotoxic insult, determining the percentage of annexin-V binding cells up to 96 h after irradiation. All six SLGC cultures examined showed either no or only marginal apoptosis (Figure 2A and 2B) even after doses as high as 10 Gy. Differentiated FBS cultures usually exhibited significantly more apoptosis than the corresponding SLGC cultures, particularly after single doses of 5 or 10 Gy, but substantially higher apoptosis was found only in the FBS culture of GBM8 (Figure 2A).

For GBM4, GBM10, and GBM22, we could not detect significantly higher apoptosis in the FBS cultures (Figure 2B). Thus, independent of the presence of EGF and FGF in longer-term cultures, these nondifferentiated SLGCs were highly apoptosis-resistant in the first 96 h after irradiation even after doses as high as 10 Gy. There was no general correlation between apoptosis in the first 96 h after irradiation and proliferation of the various cultures analyzed (data not shown). To avoid missing apoptosis very early after irradiation, we determined annexin-V binding 6, 14, and 24 h after radiation, but detected no apoptosis thereby in any culture analyzed (data not shown).

### DNA damage responses

DNA DSBs - the major lethal lesion induced by γIR - can be assessed by visualizing histone H2AX phosphorylation at serine 139 (γH2AX). Lower γIR-induced γH2AX signals have been reported in CSC-like cells compared to non-CSCs at 24 h (residual signal) but not early after irradiation [6], or early (15 min - 2 h) post-irradiation [7,8,13]. However, compared to established glioma lines either no differences [19,20] or higher γH2AX signals [18] were reported.

**Figure 3:**
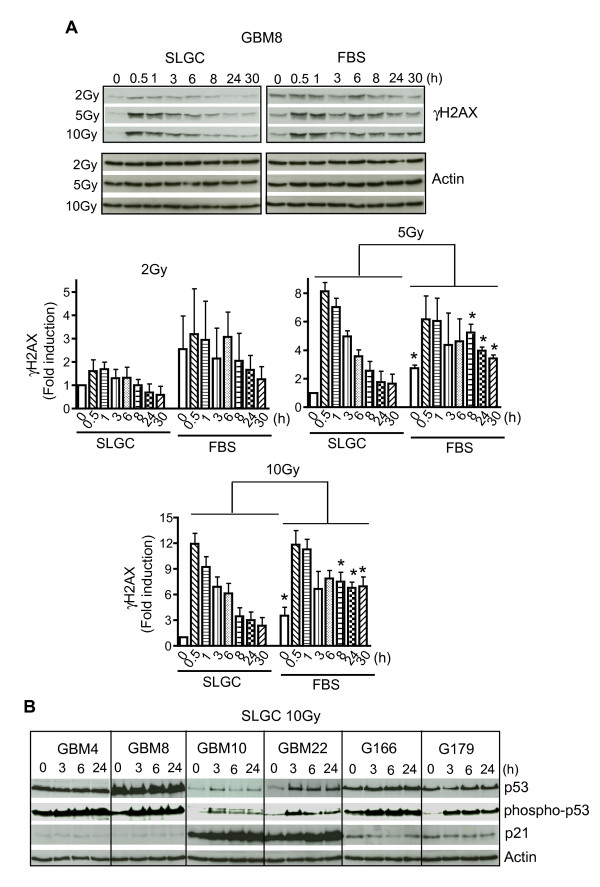
**Kinetics of expression of **γ**IR-induced DNA damage response proteins**. SLGCs and directly derived FBS cultures were irradiated with the doses indicated. **A**. Expression levels of phosphorylated histone H2AX (γH2AX) detected by Western blot (representative result, upper panel) and quantification of γH2AX-signals using Image Quant TL (lower panels). **B**. Expression levels of p53 and its target gene p21 (WAF1/Cip1), as well as of phosphorylated p53 in cell lysates collected at the indicated times after irradiation. Actin levels are shown as control. In A and B, blots shown are representative of three independent experiments.

We have made detailed kinetic analyses of γIR-induced γH2AX signals. As shown in Figure 3A, GBM8 SLGCs displayed lower baseline levels of γH2AX and a faster decline of induced signal to background than the corresponding FBS culture. For GBM4 SLGCs, which do not differentiate in FBS-containing medium, the background γH2AX-signals did not differ between the two culture types. Nevertheless, here also the induced signal declined to background levels faster in the SLGC culture (data not shown).

Activated p53 is one of the most important regulators and executors of the DNA damage response, and blocked apoptosis is often related to problems in p53 activation [15]. We therefore analyzed p53 levels at several time points after γ-irradiation. As shown in Figure 3B, radiation-induced stabilization of p53 and induction of the p53 target cyclin-dependent kinase inhibitor p21 was only observed for GBM10 and GBM22. The other 4 lines lack functional p53, showing high basal p53 expression that could not be augmented by radiation and no radiation-induced upregulation of p21. However, p53 was phosphorylated at Ser15, a step known to occur in response to radiation-mediated DNA damage.

### Influence of cytokines EGF and FGF-2 on DNA damage signals and radiation-induced apoptosis

Using inhibitor experiments, EGF and FGF-2 signaling have both been shown to affect DSB repair, cell survival and apoptosis, as well as cellular resistance to γIR [31-34]. We therefore assessed whether direct (stemness-unrelated) effects of EGF and FGF-2 contribute to observed differences in γH2AX signals between stem-like and differentiated cells under otherwise identical culture conditions.

**Figure 4:**
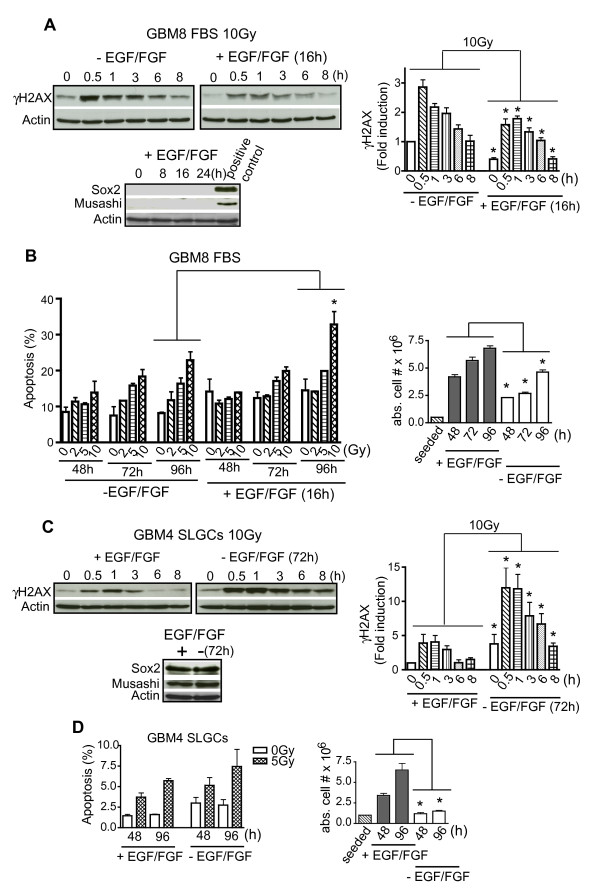
**EGF/FGF-dependence of **γ**IR-induced H2AX-phosphorylation and of apoptosis**. **A**. Differentiated GBM8 FBS cultures pretreated or not for 16 h with EGF plus FGF-2 were irradiated with 10 Gy and analyzed for γH2AX levels by Western blotting (upper left); quantification using Image Quant TL (upper right). Expression levels of the stem-cell and progenitor markers Sox2 and musashi were determined to assess the differentiation status of the cells (lower). **B**. GBM8 FBS cultures treated as in A were analyzed for apoptosis (left) and proliferation (right). **C**. GBM4 SLGCs either treated or not with EGF plus FGF-2 for 72 h were irradiated with 10 Gy and analyzed for γH2AX-levels by Western blotting (upper left); quantification (upper right). Expression levels of the stem- and progenitor markers Sox2 and musashi were determined to assess the differentiation status of the cells (lower). **D**. GBM4 SLGCs treated as in C were analyzed for apoptosis (left) and proliferation (right). Representative Western blots are presented. In the other graphs, the mean ± S.D. of three experiments is shown; asterisk, = p < .05.

As shown in Figure 4A, short-term (16 h) preincubation of differentiated GBM8 cells with EGF plus FGF-2, which did not induce either of the stemness markers Sox2 and musashi, indeed strongly reduced radiation-induced γH2AX-levels. Consistent with this, expression of phospho-DNA-PK, the key enzyme in nonhomologous end-joining, the predominant process in DSB repair [31], was increased (Additional file 2). Despite the strong reduction of γH2AX and the generally assumed anti-apoptotic nature of EGF and FGF-2 [31-34], acute addition of these two cytokines did not reduce but even tended to enhance IR-induced apoptosis of differentiated glioma cells. This was associated with increased proliferation (Figure 4B). Similar results were obtained for GBM179 (data not shown).

Conversely, a 72 h (but not a 16 h) withdrawal of EGF and FGF from GBM4 SLGCs which did not differentiate under these conditions, strongly increased γIR-induced γH2AX (Figure 4C). Nevertheless, the early apoptosis resistance of these differentiation-resistant SLGCs was not affected by the strong changes in DSB signals induced by the withdrawal of EGF and FGF (Figure 4D).

The specificity of recombinant EGF and FGF-2 and the role of EGF/FGF-2 signaling in DNA damage repair were confirmed by experiments with antibodies blocking the ligand-binding domain of EGF receptor (EGFR) and the main receptor of FGF-2 (wich is FGFR-1, [35]). As shown in Additional file 3, the two antibodies indeed abolished the cytokine-mediated decrease of γIR-induced γH2AX in differentiated GBM8 cells. However, the 72 h 12 addition of the two receptor-blocking antibodies to GBM4 SLGCs was toxic to the cells, making experiments on radiation-induced DNA damage/repair impossible under these conditions.

### γ**IR-induced mitotic catastrophe and delayed cell death in SLGC cultures**

**Figure 5:**
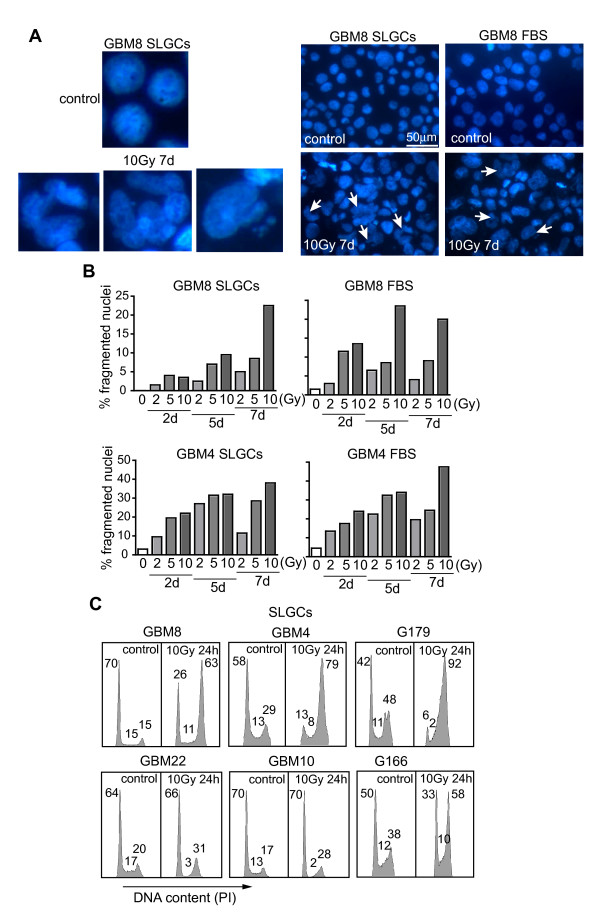
**γ IR-induced mitotic catastrophe and cell cycle arrest in SLGCs**. SLGCs and FBS cultures were irradiated with 0, 2, 5, or 10 Gy, and DAPI-stained cells with signs of mitotic catastrophe (micro- and multinucleated cells) were detected microscopically (**A**) and counted (**B**) at the time points indicated. **C**. γIR-induced cell cycle arrests in SLGC cultures. Exponentially growing cells were irradiated with 10 Gy and 24 h later cell cycle analysis was performed by flow cytometry. The percentages of cells in the different cell cycle phases are indicated. In each panel, one of two experiments with similar results is shown.

Although mitotic catastrophe is a known major cause of cell death in radio- or chemotherapy [15-17], mitotic catastrophe has so far not been explicitly assessed in studies on CSC radio- or chemosensitivity. We found considerable numbers of cells with signs of mitotic catastrophe (large micro- or multinucleated cells) in three of the six SLGC lines analyzed (GBM4, GBM8; Figure 5A, B) and G179 (not shown) already at early time points where the SLGCs showed either no or only very little apoptosis (within the first 96 h postirradiation). However, the maximum of mitotic catastrophe was later than 96 h. Particularly at high doses (10 Gy), numbers of cells with signs of mitotic catastrophe were usually higher after 7 d than after 5 d (Figure 5B). Consistent with the kinetics of appearance of multi- and micronucleated cells, increased proportions of polyploid cells were found several days (e.g., d5) after irradiation (Additional file 4). The SLGC lines undergoing mitotic catastrophe arrested in G2M after irradiation (Figure 5C). However, irradiated G166 SLGCs, despite a G2M arrest, showed no morphological signs of mitotic catastrophe. The very low level of γIR-induced mitotic catastrophe in GBM10 and GBM22 SLGCs was associated with a strong γIR-induced G1 arrest (significant decrease of S-phase cells in the first cell cycle after irradiation).

**Figure 6:**
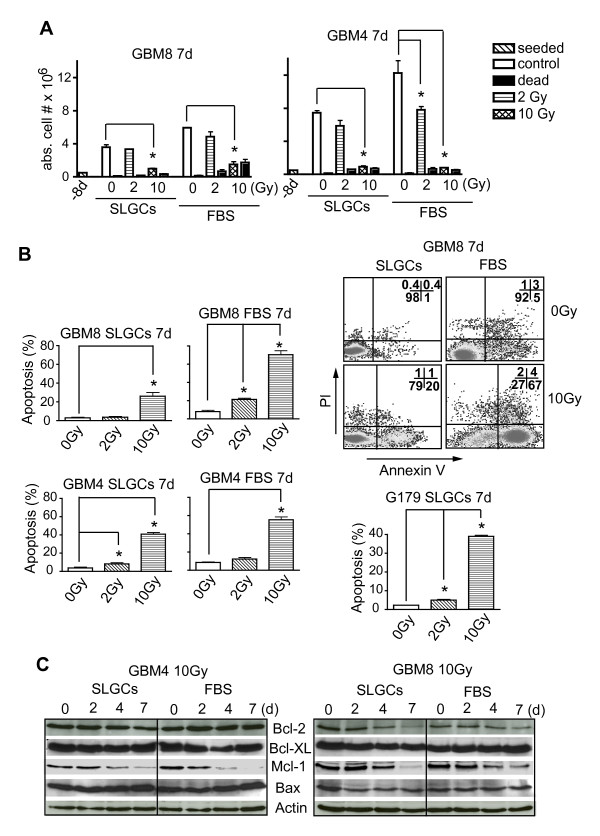
**Delayed **γ**IR-induced apoptosis in SLGCs**. SLGCs were irradiated with 0, 2, or 10 Gy. **A**. 7 d later, the numbers of viable and dead cells were counted after staining with trypan blue. **B**. Apoptosis was assessed flow cytometrically after staining with Annexin V/PI. A representative flow cytometry analysis is shown (upper right). **C**. Kinetics of expression of pro- and anti-apoptotic proteins were assessed by Western blotting.

Most cells undergoing mitotic catastrophe are destined to die. Seven days after irradiation, we found a clear reduction in viable cell numbers and an increase in dead cells not only in the FBS- but also in the SLGC cultures of GBM4, GBM8 (Figure 6A) and G179 (not shown), particularly at higher doses. Most of these cells died by delayed apoptosis. This is suggested by the kinetics of annexin-V exposure (see Figure 2 and 6B), and the downregulation of the anti-apoptotic protein Mcl-1 (Figure 6C), which correlated well with each other. The three SLGC lines GBM10, GBM22, and G166 did not show substantial late apoptosis (Additional File 5). In accord with less early/late apoptosis and less mitotic catastrophe, GBM8 SLGCs in clonogenic assays were also less sensitive to radiation than the corresponding FBS-differentiated cells (Additional file 6). Consistent with the *in vitro *results on mitotic catastrophe and delayed apoptosis, a 10-Gy irradiation completely abolished the tumorigenicity of GBM4 SLGCs in immunocompromised mice (data not shown).

**Figure 7:**
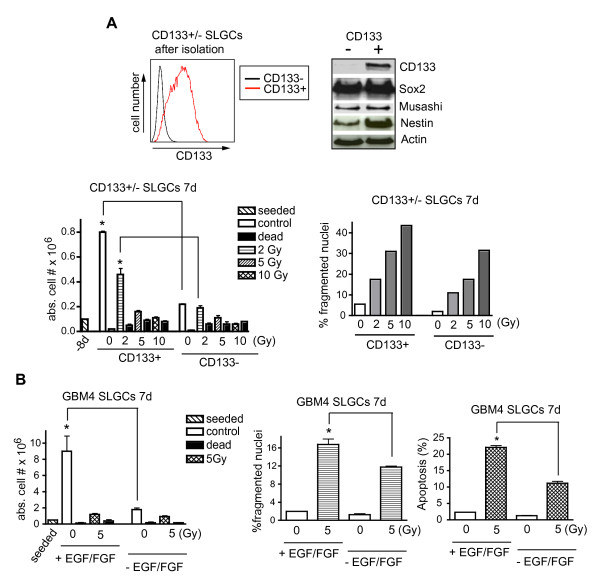
**Correlation of proliferation with **γ**IR-induced mitotic catastrophe and late apoptosis in SLGCs**. **A**. Analyses of CD133+ and CD133- immature glioma cells. Flow cytometry analysis of CD133 expression in magnetic bead-separated CD133+/CD133-populations of GBM8 SLGCs (upper left). Expression of CD133, Sox2, musashi and nestin as determined by Western blotting (upper right). Viable and dead cells (lower left) and cells with signs of mitotic catastrophe (lower right) 7 d after irradiation. **B**. Influence of EGF plus FGF-2 on proliferation, mitotic catastrophe, and apoptosis of GBM4 SLGCs. The cells were incubated with or without EGF plus FGF-2 (20 ng/ml). After 24 h the cells were irradiated, and 7 d later analyzed. In the cytokine-treated cultures, EGF and FGF-2 were replenished after 3 days. Mean ± S.D. of at least three experiments is shown; statistical significance (p < .05).

Also purified CD133+ GBM8 SLGCs (Figure 7A) showed clear, dose-dependent signs of mitotic catastrophe relatively late after γIR (e.g., d7). They showed even more mitotic catastrophe than undifferentiated (Sox2+ and musashi+) CD133- cells or unseparated undifferentiated cells (see Figure 5B). Consistently, CD133+ SLGCs proliferated considerably faster than the undifferentiated CD133- cells (Figure 7A lower left).

The data thus far obtained suggested that the rate of proliferation influences the extent of late IR-induced apoptosis of SLGCs, particularly of those with nonfunctional p53 which undergo mitotic catastrophe after γ-irradiation. Indeed, when we treated GBM4 SLGCs with EGF plus FGF-2 (or not) and assessed cell numbers, mitotic catastrophe and apoptosis 7 days after a 5 Gy irradiation, a correlation among the three parameters was observed (Figure 7B). Similar results were obtained with GBM8 SLGCs treated with 20 ng/ml EGF/FGF (vs. 5) (data not shown).

## Discussion

Glioblastomas are among the most aggressive tumors, being incurable to date [36] and CSCs are thought to have a major impact on their therapy resistance [4-6]. Independent of tumor entity, CSCs are generally thought to be relatively radio- and chemotherapy-resistant, but, regarding cell death modalities, investigations have thus far concentrated on apoptosis occurring early after the genotoxic insult [6,9,10,12]. We confirmed here that primary SLGCs are extraordinarily resistant to apoptosis within the first few days after γ-irradiation (up to 96 h and up to doses as high as 10 Gy in this study), but we also show that at such doses, SLGCs very late (>4 days after irradiation) can undergo apoptosis. However, this late apoptosis was restricted to SLGCs undergoing G2M arrest resulting in mitotic catastrophe and seems thus to be restricted to proliferating cells. All these SLGC lines had nonfunctional p53. However, one line with nonfunctional p53 which underwent IR-induced G2M arrest did not undergo mitotic catastrophe or late apoptosis. Mitotic catastrophe thus seems to be an absolute requirement for this late type of cell death. FBS-differentiated cells tended to show significant apoptosis already between 48 and 96 h post-irradiation. Differences in apoptosis early after irradiation did not strictly correlate with observed differences in initial or residual γH2AX. In addition, the γH2AX levels turned out to depend strongly on the presence of the CSC-culture cytokines EGF plus FGF, confounding *in vitro *analyses of DNA damage and repair.

We have for the first time compared radiation responses of primary stem-like glioma cells with that of more differentiated cells directly derived from the former. So far, similar studies have either compared CSC surface marker-positive with surface marker-negative cells [6,8,11,13], traditional cell lines cultured as spheres or adherently in FBS [7], or else patient-derived CSC-like cells with traditional FBS-cultured cell lines [18,19]. In our study, only one SLGC line displayed substantial surface AC133 (the stem cell-specific glycosylation-dependent epitope of CD133). However, there is considerable evidence for AC133/CD133 negative tumor stem cells in glioblastoma [23,24].

It is generally assumed that differentiated tumor cells are more susceptible to genotoxic treatment-induced apoptosis than stem-like tumor cells. In our study, three SLGC lines (GBM8, G179, and G166) could be induced to differentiate by withdrawing EGF and FGF-2 and adding FBS. Although all these differentiated lines underwent more γIR-induced apoptosis than the corresponding SLGC cultures, a large increase was only observed for the FBS culture of one line, GBM8. An explanation for this might be that GBM8 SLGCs differentiate more readily than G179 and G166 SLGCs. GBM8 SLGCs lose Sox2 expression completely already a few days after EGF/FGF removal (not shown), whereas this process is much slower in the case of G179 and particularly G166 where the maximal (and incomplete) decrease of Sox2 expression was observed only after 3 to 4 weeks. Thus there may well be differences in the degree of differentiation among these three differentiated lines, but other mechanisms explaining the difference in the apoptotic response are also conceivable.

Cell death caused by mitotic catastrophe can occur at the first cell division after irradiation or at one of the next thereafter either as secondary apoptosis or as necrosis [14,16]. Mitotic catastrophe can be enhanced in cells lacking p53. In contrast, p53 is crucial for genotoxically induced apoptosis in cell types prone to primary apoptosis [15,37-39]. We observed here that most SGLCs with nonfunctional p53 underwent apoptosis mostly later than 4 d post-irradiation, i.e., presumably postmitotic, secondary apoptosis. Why one of the SLGC lines with nonfunctional p53 and γIR-induced G2M arrest failed to undergo mitotic catastrophe and late apoptosis is unclear at the moment and will be the subject of further studies in our laboratory.

Many primary glioblastomas are wild-type for p53 [40]. However, in line with previous studies on primary GBM [41] we found that p53 is not functional in most (4/6) primary glioblastomas studied by us. A possible reason for the lacking p53 stabilization is the deletions or mutations in PTEN frequently associated with primary GBM, since PTEN has been described as important for p53 stabilization [10,42]. Other possible causes are mdm2 gene amplification, loss of p14^ARF ^[43] or overexpression of the NF-κB-signaling component RIP1 [44], all frequently associated with GBM. Interestingly, the γIR-induced phosphorylation of p53 at serine 15 was intact, presumably because of an intact ataxia teleangiectasia-mutated pathway.

The non-differentiating FBS cultures of GBM4, GBM22 and GBM10 were almost completely apoptosis-resistant in the first 4 d after irradiation despite the prolonged γH2AX signals and the week-long absence of EGF and FGF-2. This early apoptosis resistance thus seems to be independent of potential direct anti-apoptotic effects of EGF and FGF-2 [31-34]. In line with this, Bao *et al*. described that, 20 h after irradiation, CD133+ glioblastoma cells showed apoptosis resistance irrespective of whether EGF and FGF were present after irradiation [6]. However, immediately after cytokine withdrawal, anti-apoptotic and DNA-repair proteins directly induced by EGF/FGF may still be operating as we have observed differences in γH2AX after cytokine withdrawal for 72 h but not for 16 h. Thus, early after cytokine withdrawal, direct cytokine effects and stem cell-intrinsic effects on cell survival and DNA damage signals are not yet discernible.

It has so far not been studied whether EGF and FGF-2 confound comparisons of DNA damage signals between stem-like and non-stem-like cells. We observed that acute EGF/FGF addition to differentiated tumor cells reduces IR-induced γH2AX-signals while subacute and chronic withdrawal from non-differentiating stem-like cells increases them. This implies that, due to the different cytokine requirements, *in vitro *studies on previously suggested intrinsic differences in γIR-induced DNA-damage [7,13] and repair [6] between CSCs and non-CSCs are problematic. Thus, a strict *in vivo *comparison would be best, but comprehensive experiments on precisely defined cell populations will hardly be possible. Very recently, two other groups reported that EGF [45] and FGF-2 [46] activate DNA damage/repair as reflected by reduced γIR-induced γH2AX signals. In these studies, conventional bronchial carcinoma cell lines [45] or primary keratinocyte progenitor cells [46] were used. The results from these studies support our findings.

We have observed that acute EGF/FGF-2 addition to differentiated cultures of p53-deficient SLGCs even enhanced γIR-induced apoptosis rather than decreasing it, despite the usually assumed pro-survival effects of EGF and FGF-2 and even though the γH2AX signals were reduced by the addition of the two cytokines. However, proliferation was accelerated, indicating a stronger correlation of the observed delayed γIR-induced apoptosis to cellular proliferation than to DNA damage assessed by γH2AX. We similarly examined two p53-deficient SLGC lines and, depending on the concentration of the two stem-cell mitogens, there was a correlation of cellular proliferation with mitotic catastrophe and late apoptosis induced by 5 or 10 Gy irradiation. However, this observation may only apply to tumor lines whose postirradiation survival is independent of EGF and FGF.

Mitotic catastrophe is associated with proliferation. It is often argued that CSCs, like many types of normal tissue stem cells, may be relatively quiescent, but it has been shown that even some normal tissue stem cells proliferate very fast *in vivo *[47]. Moreover, a recent study showed that in some GBM patients CD133+ glioblastoma cells coexpress the proliferation marker Ki67 [48]. In addition, in cultures of normal neural stem cells, CD133 is expressed on the surface of proliferating cells [49]. We have made similar observations in the CD133+ SLGC line examined by us, and accordingly found even more IR-induced mitotic catastrophe among bead-purified CD133+ cells than in the CD133- immature cells of the same tumor. It is however unclear at the moment whether this observation can be generalized.

Recent publications showed that transient pretreatment with a proliferation-inducing agent leads to the effective chemotherapy-mediated elimination of dormant hematopoietic stem cells and human primary leukemia stem cells *in vivo *[50,51], but so far it has not been determined whether this also applies to genotoxically treated solid tumor stem cells. Our results suggest that this might only apply to those CSCs which undergo genotoxic treatment-induced mitotic catastrophe. Besides a search for suitable proliferation-inducing agents, more research is necessary regarding the proliferation status of CSCs and cell death associated with mitotic catastrophe in cancer patients undergoing genotoxic therapies.

## Abbreviations used

IR: ionizing radiation; EGF: epidermal growth factor; FGF: fibroblast growth factor; CSCs: cancer stem cells; SLGCs: stem-like glioma cells; ECM: extracellular matrix proteins; DSBs: DNA-double strand breaks; R: receptor; PI: propidium iodide.

## Competing interests

The authors declare that they have no competing interests. NE is an employee of Proquinase, but Proquinase did not sponsor the study.

## Authors' contributions

EF performed most of the experiments and contributed to writing the manuscript. SG, CT, and NE performed experiments. AW collected the tumor material and provided clinical information. GN was the Principal Investigator of the study and largely wrote the manuscript. All authors read and approved the final manuscript.

## Supplementary Material

Additional file 1 **Expression levels of CD133 in SLGC cultures determined by flow cytometry**. Traces of AC133/CD133 could be detected in lysates of GBM22 and G179 SLGCs by Western blot (not shown). Click here for file

Additional file 2 **Expression levels of phospho-DNA-PK and total DNA-PK in GBM8 FBS cultures**. The cultures were supplemented or not with EGF/FGF-2 for 16 h and irradiated with 10 Gy thereafter. Click here for file

Additional file 3 **Analysis of EGF/FGF-dependent modulation of **γ**H2AX expression in the presence of receptor-blocking antibodies**. **A**. Differentiated GBM8 FBS cultures pretreated for 16 h with EGF plus FGF-2 or not were irradiated with 10 Gy and analyzed for γH2AX levels by Western blotting. Receptor blocking antibodies abolished the cytokine-mediated decrease of γIR-induced γH2AX levels. **B**. GBM4 SLGCs either treated standardly with EGF plus FGF-2 or not for 72 h were irradiated with 10 Gy and analyzed for γH2AX levels by Western blotting. Receptor blocking antibodies strongly increased the basal γH2AX level in non-irradiated cells. Since this was accompanied by induction of cell death (not shown), the increased γH2AX is most likely due to apoptotic DNA damage [52], thus rendering analyses of γIR-induced DNA damage impossible under these conditions. Click here for file

Additional file 4 **Determination of polyploid cells in irradiated SLGCs**. SLGCs were irradiated with 10 Gy and cell cycle analysis was performed at d5 after irradiation. Mean ± S.D. of at least three experiments is shown; statistical significance (p < .05). Click here for file

Additional file 5 **SLGCs not undergoing late **γ**IR-induced apoptosis**. SLGCs were irradiated with the doses indicated and apoptosis was assessed by flow cytometry after 7 d. Mean ± S.D. of at least three experiments is shown; statistical significance (p < .05). Click here for file

Additional file 6 **Survival curves of GBM8 and GBM4 SLGCs and corresponding FBS cultures determined by clonogenic assay**. Cells were seeded and then irradiated 6 h later at the doses indicated. After 10 d (FBS cultures) or 20 d (SLGC cultures), colonies were fixed and stained with 0.5% crystal violet. Experiments were performed in triplicates. Click here for file
