# Supplementary figures and images for: Delayed cell death associated with mitotic catastrophe in γ-irradiated stem-like glioma cells
Source: Radiat Oncol. 2011 Jun 10;6:71. doi: 10.1186/1748-717X-6-71 (PMC3130665; doi:10.1186/1748-717X-6-71)

# Additional file 1

CD133 *in vitro*

SLGCs

FBS

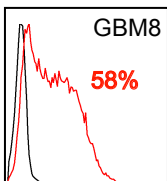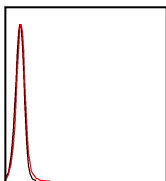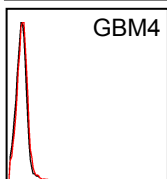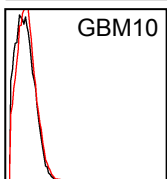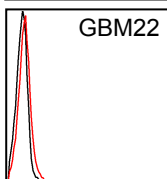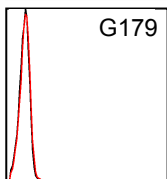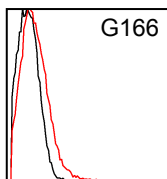

— CD133  
— negative

cell number ↑

CD133 →

Supplement: Additional file 1 — Expression levels of CD133 in SLGC cultures determined by flow cytometry. Traces of AC133/CD133 could be detected in lysates of GBM22 and G179 SLGCs by Western blot (not shown). [file 1748-717X-6-71-S1.PDF]

## Additional file 2

GBM8 FBS 10Gy

+ EGF/FGF (16h)

0 0.5 1 3 0 0.5 1 3 (h)

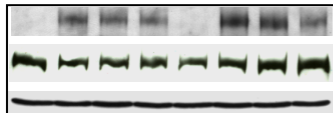

phospho-DNA-PK

DNA-PK

Actin

Supplement: Additional file 2 — Expression levels of phospho-DNA-PK and total DNA-PK in GBM8 FBS cultures. The cultures were supplemented or not with EGF/FGF-2 for 16 h and irradiated with 10 Gy thereafter. [file 1748-717X-6-71-S2.PDF]

## Additional file 3

**A**

### GBM8 FBS

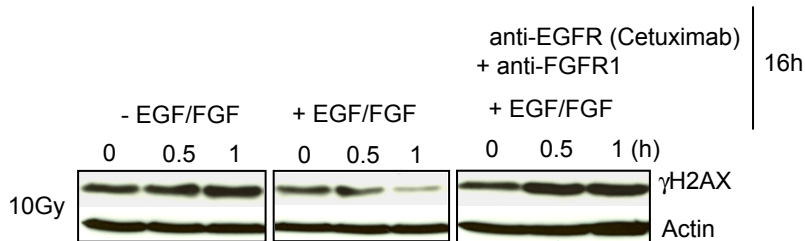

**B**

### GBM4 SLGC

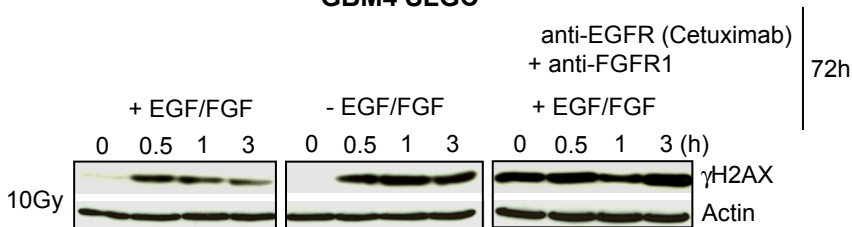

Supplement: Additional file 3 — Analysis of EGF/FGF-dependent modulation of γH2AX expression in the presence of receptor-blocking antibodies. A. Differentiated GBM8 FBS cultures pretreated for 16 h with EGF plus FGF-2 or not were irradiated with 10 Gy and analyzed for γH2AX levels by Western blotting. Receptor blocking antibodies abolished the cytokine-mediated decrease of γIR-induced γH2AX levels. B. GBM4 SLGCs either treated standardly with EGF plus FGF-2 or not for 72 h were irradiated with 10 Gy and analyzed for γH2AX levels by Western blotting. Receptor blocking antibodies strongly increased the basal γH2AX level in non-irradiated cells. Since this was accompanied by induction of cell death (not shown), the increased γH2AX is most likely due to apoptotic DNA damage [52], thus rendering analyses of γIR-induced DNA damage impossible under these conditions. [file 1748-717X-6-71-S3.PDF]

## Additional file 4

### GBM4 SLGCs (5d)

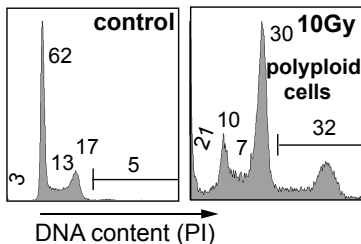

### GBM4 SLGCs (5d)

### GBM8 SLGCs (5d)

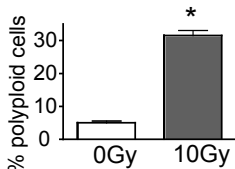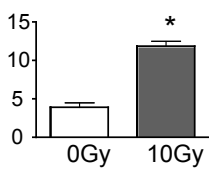

Supplement: Additional file 4 — Determination of polyploid cells in irradiated SLGCs. SLGCs were irradiated with 10 Gy and cell cycle analysis was performed at d5 after irradiation. Mean ± S.D. of at least three experiments is shown; statistical significance (p < .05). [file 1748-717X-6-71-S4.PDF]

## Additional file 5

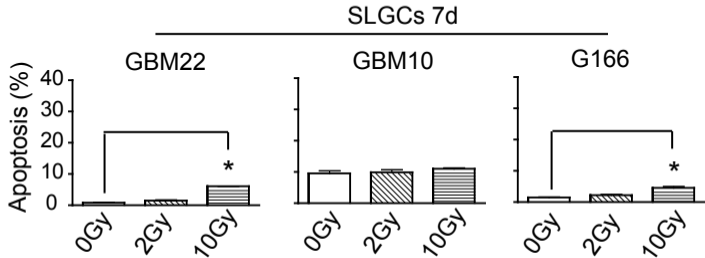

Supplement: Additional file 5 — SLGCs not undergoing late γIR-induced apoptosis. SLGCs were irradiated with the doses indicated and apoptosis was assessed by flow cytometry after 7 d. Mean ± S.D. of at least three experiments is shown; statistical significance (p < .05). [file 1748-717X-6-71-S5.PDF]

## Additional file 6

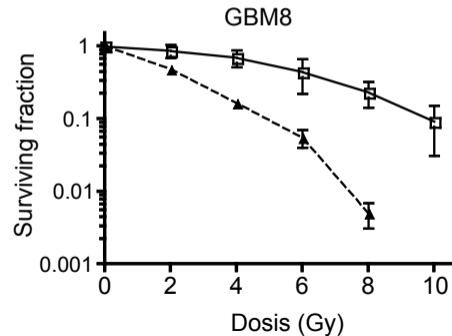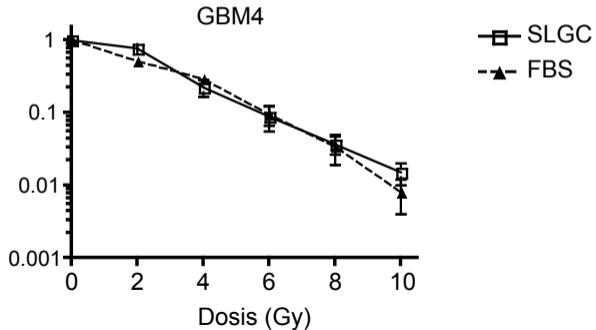

Supplement: Additional file 6 — Survival curves of GBM8 and GBM4 SLGCs and corresponding FBS cultures determined by clonogenic assay. Cells were seeded and then irradiated 6 h later at the doses indicated. After 10 d (FBS cultures) or 20 d (SLGC cultures), colonies were fixed and stained with 0.5% crystal violet. Experiments were performed in triplicates. [file 1748-717X-6-71-S6.PDF]
